# Comprehensive Retrospective Analysis of Colorectal Cancer Incidence Patterns in Saudi Arabia

**DOI:** 10.3390/life13112198

**Published:** 2023-11-11

**Authors:** Ahmed M. Basudan, Abdulrahman Mohammed Basuwdan, Manal Abudawood, Raed Farzan, Mohammad A. Alfhili

**Affiliations:** 1 Department of Clinical Laboratory Sciences, College of Applied Medical Sciences, King Saud University, Riyadh 12372, Saudi Arabiarfarzan@ksu.edu.sa (R.F.); malfeehily@ksu.edu.sa (M.A.A.); 2 College of Medicine, King Saud bin Abdulaziz University for Health Sciences (KSAU-HS), Riyadh 14611, Saudi Arabia

**Keywords:** colorectal cancer, Saudi Arabia, patterns, cancer registry, incidence, cancer trends

## Abstract

Colorectal cancer (CRC) is the commonest cancer in Saudi males and the third most common in Saudi females. Although CRC represents a major public health challenge, the resources to evaluate its burden are inadequate. This study aims to elucidate the magnitude of CRC incidence trends in the Saudi population by age, gender, and administrative region. Data for multiple incidence measures were analyzed from the Saudi Cancer Registry (SCR) retrospectively from 2001 to 2018. Temporal trends were further analyzed by age group, gender, administrative region, and globally using joinpoint regression analysis. The number of CRC cases climbed by 335.6% and the disease increased by 56.4% to comprise 12.2% of all cancers cases. The age-standardized incidence rate (ASR) increased by 152% overall, and the median age at diagnosis peaked at 60 and 58 years for males and females, respectively. Riyadh and the Eastern Region had the highest ASR for both genders, peaking at 21.8 and 19.2 for males and 17.4 and 16.5 for females per 100 K population. Our prediction model identified growing trends with annual percentage changes (APCs) of 4.59% in males (CI: 3.1–6.1) and 3.91% among females (CI: 2.4–5.5). Males above 75 years had the highest APC (7.9%, CI: 5.3–10.7), whereas the highest APC among females was found in the age group 70–74 (5.4%, CI: 2.8–8). Globally, APC was the highest for both genders compared to selected countries. CRC incidence is increasing alarmingly in Saudi Arabia and is projected to continue. There is a need for better screening strategies, preventative measures, and awareness-building.

## 1. Introduction

Colorectal cancer (CRC) is described as the atypical expansion of cells in the colon or rectum. CRC is the world’s third most frequent malignancy in both men and women, after breast cancer and lung cancer [1]. Following lung cancer, it also has recently become the second most common cause of cancer fatality worldwide [2]. Therefore, CRC is responsible for 10% and 9.4% of global cancer incidence and mortality, respectively. In 2020, 1.93 million new CRC cases and 0.94 million CRC-related deaths were detected [3]. Worldwide, CRC incidence is 3–4 times more evident in males than females, and more common in developed than in developing countries [4]. Although our understanding of CRC pathophysiology has evolved tremendously over the years, its incidence is still increasing; thus, it poses a significant public health challenge. In 2040, 3.2 million CRC cases are anticipated to be recorded globally, signifying the worldwide burden [3]. In Saudi Arabia, CRC is the commonest cancer in males and the third most common among females [5,6,7]. In 2016, there were an estimated 1659 new cases of colorectal cancer and 730 deaths from the disease in Saudi Arabia. Although CRC is a significant public health concern in the kingdom, the reports for epidemiological parameters are inadequate in comparison to numerous Western nations. Accordingly, it is imperative to deeply characterize the CRC incidence patterns in the Saudi population by gender, age group, and geographic region, among other factors. This study provides an extensive evaluation of CRC incidence measures for the period from 2001 to 2018. The outcome of this article should delineate the implications with regard to policy making and the implementation of early detection and screening approaches, with the eventual aim of cancer mortality reduction.

## 2. Materials and Methods

### 2.1. Acquisition of Data

The National Health Information Center (NHIC) hosts data for the Saudi Cancer Registry (SCR), which reports to the Saudi Health Council (SHC). The SCR oversees the collection of information through five regional offices that report to a main office, in order to offer extensive oversight of all healthcare institutions within the kingdom. The retrospective assessment of records spanning January 2001 through December 2018 was conducted. At the time of writing this manuscript, there were no publicly available data for any years following 2018. Saudi male and female patients with colorectal cancer across all age categories and administrative regions were analyzed, and pertinent data, including descriptive features and other statistical parameters, were reported. Cases with unknown nationalities and/or unknown International Classification of Diseases (ICD) codes were omitted from the analyses (Supplementary Table S1). The collected data cover each of Saudi Arabia’s 13 administrative regions, which include Asir, Baha, the Eastern Region, Hail, Jazan, Jouf, Madinah, Makkah, Najran, Northern Borders, Qassim, Riyadh, and Tabuk.

### 2.2. Statistical Parameters

The incidence statistical parameters were all reported in accordance with the data provided by the SCR. Every incidence rate was represented as a percentage of 100,000 populations.

The parameters were calculated as follows:

Age-specific incidence rate (AIR):

$$AIR=\frac{No.of\;cancer\;cases\;occurring\;during\;a\;specific\;period\;in\;apopulation\;of\;a\;specific\;age\;group}{No.of\;midyear\;population\;of\;that\;age\;group}$$

Age-standardized incidence rate (ASR):

$$ASR=\frac{Total\;no.of\;expected\;cases}{Total\;standard\;poulation\;size}$$

The ASR is a concise evaluation of the rate that would exist in a population if it had a typical (reference) age structure. In this particular case, the World Standardized Population was used as reference.

A Shapiro–Wilk normality test was used to determine whether the data had a normal distribution or not. A two-sided t test was performed for two-group comparisons after confirmation of normality. A 95 percent confidence level was used, and *p* values less than 0.05 were considered significant.

### 2.3. Temporal Trend Estimation 

The Joinpoint regression software (version 5.0.1 [8]), which assesses shifting linear trends across subsequent segments of time, was used to forecast temporal trends for age-standardized rates. The models used had limited joinpoints in order to decrease the risk of excessive diversity in how trends are reported over time. To determine whether the changes were statistically different from zero, a Z test was utilized. Trends were reported as the annual percentage change (APC) with a 95% confidence interval (CI) and a significance level of alpha = 0.05. The output of this modeling analysis can be considered a summary of incidence rates over the specified period of time. Positive values indicate increasing trends, while values below zero imply decreasing trends. The methodology has been frequently employed to assess shifts in incidence trends [9,10,11,12,13,14].

## 3. Results

### 3.1. Total Number of Cancer Cases in Saudi Arabia

**Figure 1:**
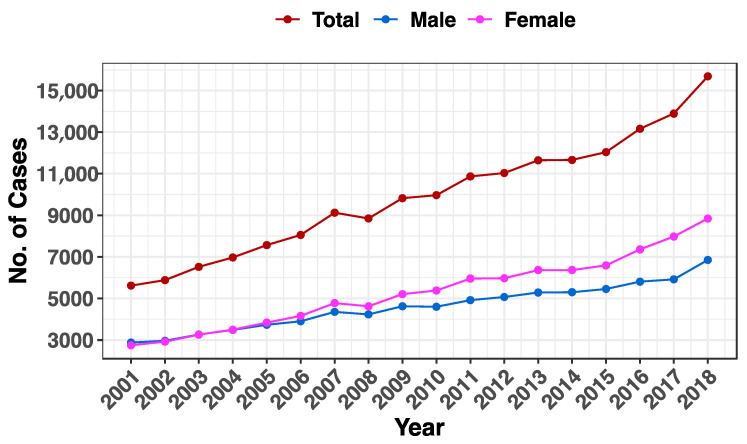
A breakdown of cancer cases in Saudi Arabia by year. The y-axis displays the total number of cases of all cancers from 2001 to 2018 (x-axis) for all genders (brown), males (blue), and females (pink).

The overall number of cases of cancer grew by 179.3% for both genders collectively, from 5616 cases in 2001 to 15688 cases, in 2018. For males only, 2875 and 6848 cases were reported for that period, respectively, which represents a 138.2% increase. The increase was higher among females, where 2741 cases were recorded in 2001 and reached 8840 cases in 2018, leading to a 222.5% increase in the number of the cases of all cancers (Figure 1 and Table 1).

### 3.2. Number of Cases and Age at Diagnosis for Colorectal Cancer in Saudi Arabia

**Figure 2:**
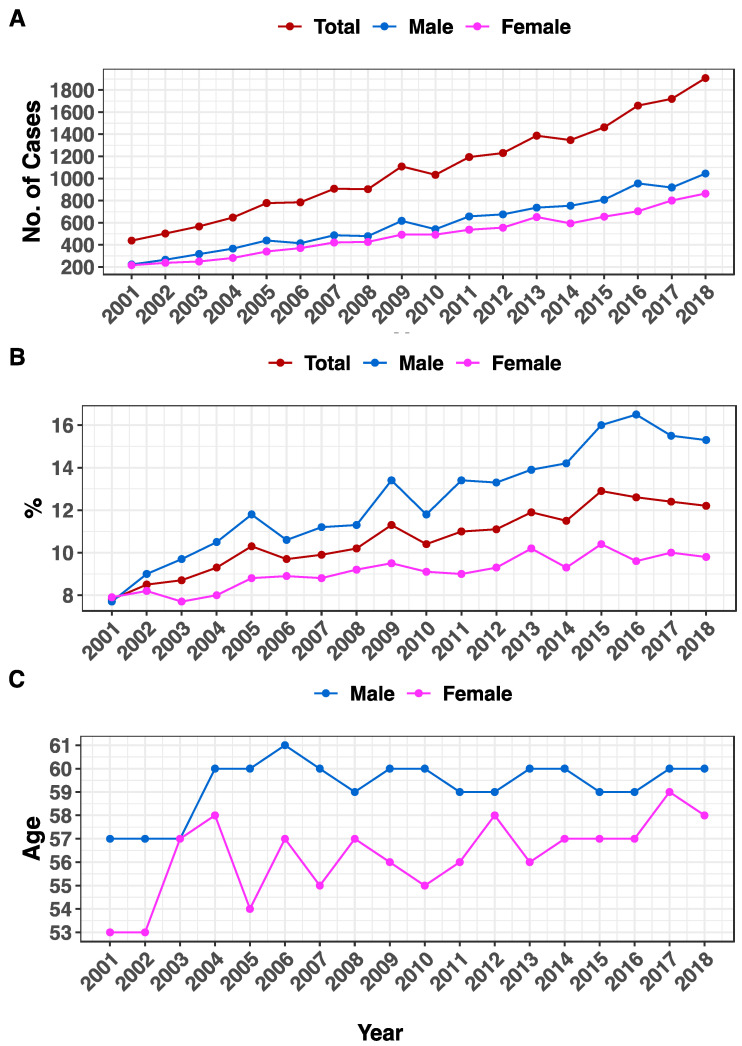
Number of cases and age at diagnosis for CRC in Saudi Arabia. (**A**) The number of CRC cases (y-axis) among Saudi citizens. (**B**) The percentage of CRC cases (y-axis) of all cancers. (**C**) The y-axis reflects the median age at CRC diagnosis. The years from 2001 to 2018 are represented on the x-axis. Genders are color-coded (total = brown, males = blue, and females = pink).

For colorectal cancer specifically, there were 335.6% more cases among Saudis, increasing from 438 cases in 2001 to 1908 in 2018 (Table 1 and Figure 2A). Throughout that period, colorectal cancer for both genders increased by 56.4% from 7.8% in 2001 to comprise 12.2% of all cancer cases in 2018. The increase was more pronounced in males compared to females (98% vs. 24.1%, Figure 2B and Table 1). In 2001, the median age at diagnosis for males was 57; then, it kept fluctuating to reach 60 years in 2018 (5.3% increase). For females, the increase was 9.4%, from 53 years in 2001 to 58 years in 2018 (Figure 2C and Table 1). 

### 3.3. CRC Incidence Rates in the Saudi Population

**Figure 3:**
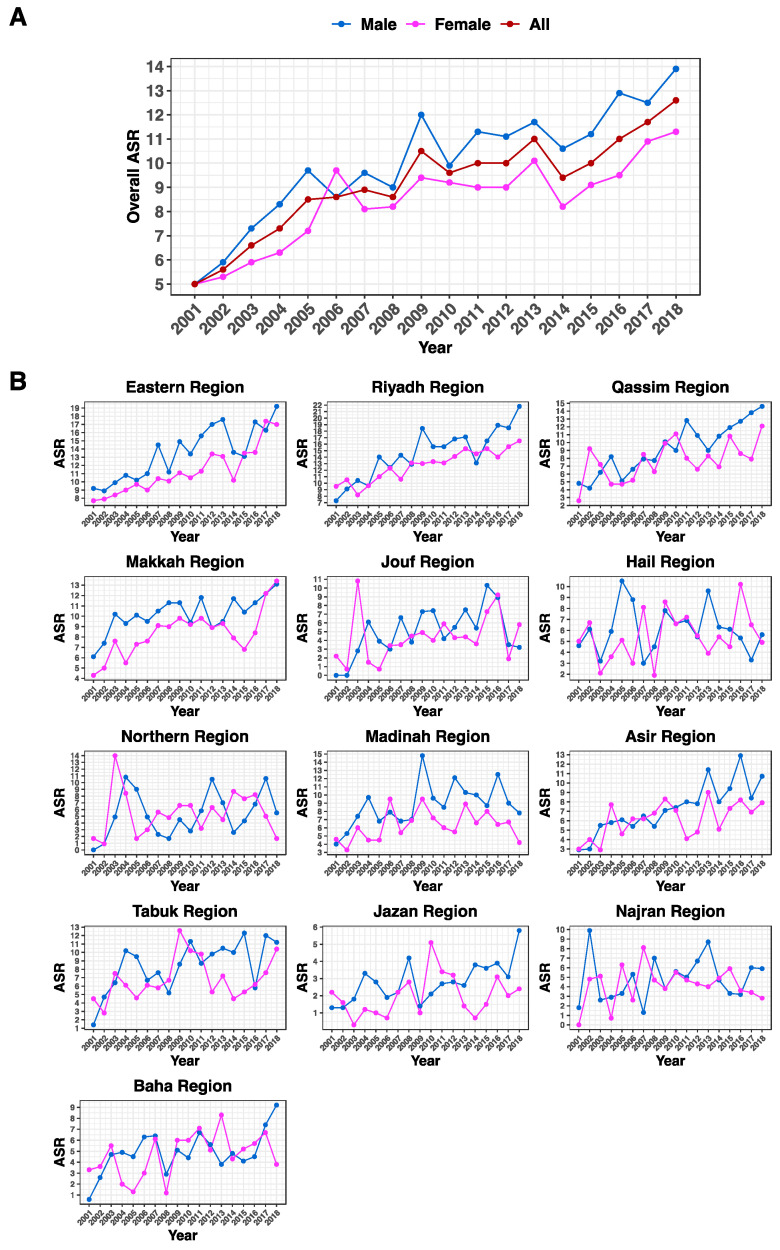
Age-standardized incidence rate (ASR) for CRC among the Saudi population. (**A**) Overall ASR for CRC in the whole country for all gender groups. (**B**) ASR broken down by each administrative region in Saudi Arabia. Genders are defined by color (total = brown, males = blue, and females = pink). The years 2001 through 2018 are specified on the x-axes. All ASR rates indicated on the y-axes are per 100,000 population.

Between 2001 and 2018, the colorectal cancer overall ASR had an overall increase of 152% for both genders, 178% for males, and 126% for females (Figure 3A and Table 1). The dissection of ASR by geographical region resulted in multiple findings. While some regions showed fluctuations during that period, Riyadh and the Eastern Region noticeably had the highest ASR among males, which peaked at 21.8 and 19.2 per 100,000 population, respectively (Figure 3B). The same regions also had the highest ASR among females.

**Figure 4:**
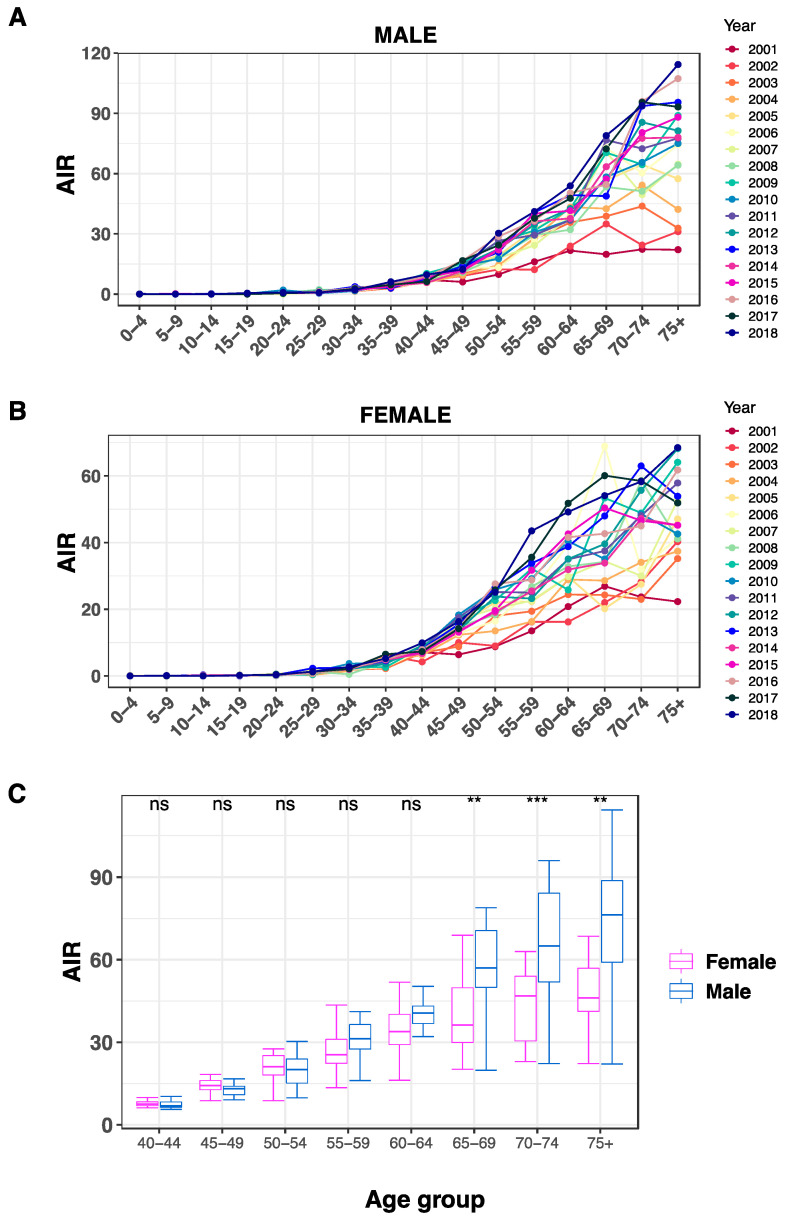
AIR for CRC among the Saudi population. AIR values are plotted for each age group (x-axis) among males (**A**) and females (**B**). (**C**) Boxplot comparisons of AIR between males (blue) and females (pink) for each age group. ns indicates no significance, ** *p* < 0.01, and *** *p* < 0.001. All AIR rates indicated on the y-axes are per 100,000 population.

Even though AIR fluctuated throughout the years. During that time frame, AIR nearly continuously achieved its highest point after 70 years of age (Figure 4A,B). The sole exception for males was in 2007, where the highest point was in the 65–69 age group. For females, the exceptions were in the years 2001, 2006, 2015, and 2017, with peaks in the 65–69 age group. In addition, comparisons of AIR between males and females for each age group revealed significantly higher AIR in males for the age groups 65–69, 70–74, and 75+, as indicated in Figure 4C (*p* < 0.05).

### 3.4. Trends of Colorectal Cancer Incidence in Saudi Arabia

**Figure 5:**
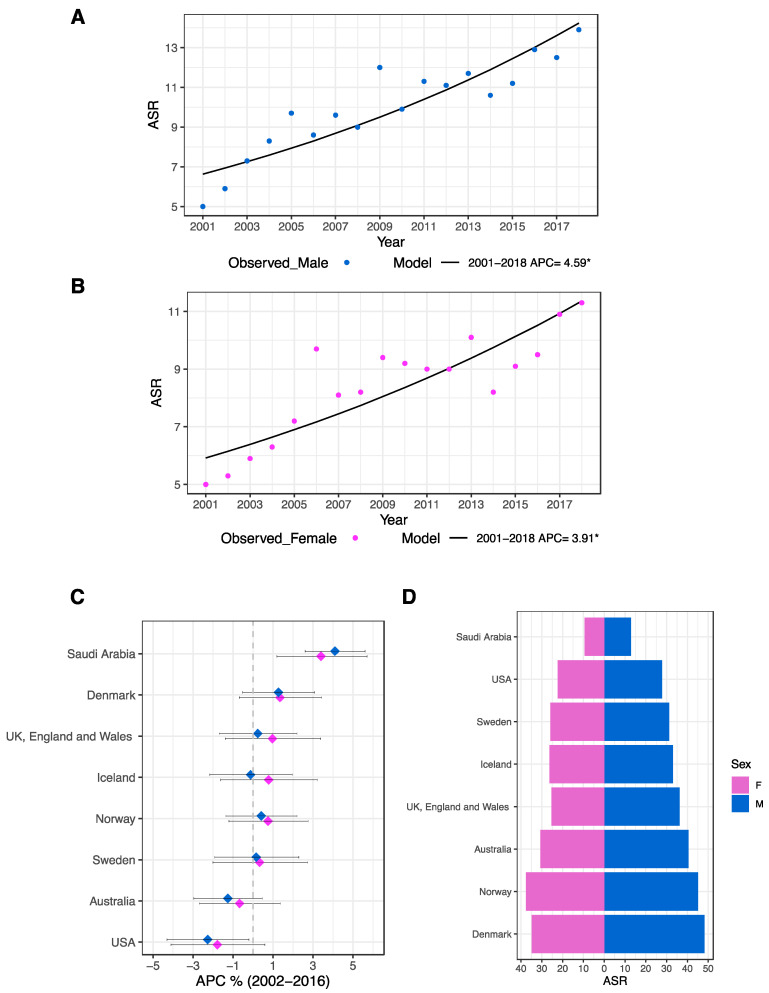
Trends in CRC incidence in Saudi Arabia. Regression analysis of CRC overall ASR in the Saudi male (**A**) and female (**B**) populations from 2001 to 2018. The estimated APCs of the prediction models are shown underneath each plot (* denotes *p* < 0.05). (**C**) The APC of CRC ASR for designated nations in contrast to Saudi Arabia (2002–2016). The middle diamond represents the estimated APC value, while the horizontal line represents the 95% CI. (**D**) Pyramid plot for CRC ASR in Saudi Arabia and selected countries in 2016 for both genders. Data for the selected countries in (**C**,**D**) were obtained through the International Agency for Research on Cancer (IARC) [15].

To predict the trends of colorectal cancer for the years 2001 through 2018, a joinpoint regression analysis was performed for the age-standardized incidence rates (ASR) of both genders. In the male cohort, the model revealed rising trends with an annual percentage change (APC) of 4.59% (CI: 3.1–6.1, *p* < 0.05, Figure 5A). For females, the APC also increased by 3.91% (CI: 2.4–5.5, *p* < 0.05, Figure 5B). 

APC levels in males were highest in the age group above 75 (7.9%, CI: 5.3–10.7, *p* < 0.05), whereas APC levels in females were highest in the age group of 70–74 (5.4%, CI: 2.8–8, *p* < 0.05), according to an age-specific analysis that was also carried out. The values for all age groups by gender are illustrated in Table 2. 

In addition, region-specific trends were calculated and revealed that the regions of Asir (6.8%), Qassim (6.6%), Baha (6.2%), and Tabuk (6%) had the highest APC among males. On the other hand, the regions of Jouf (6.9%), Qassim (4.4%), East (4.3%), Makkah (4.1%), and Asir (4%) had the highest among females. Detailed values for all administrative regions by gender are given in Table 3.

Globally, the calculated ASR-APCs of colorectal cancer for multiple countries were derived for comparison with Saudi Arabia for the time period of 2002–2016 [15]. In Saudi Arabia, our examination revealed APCs of 4.1% (CI 2.6–5.6, *p* < 0.05) and 3.4% (1.2–5.7, *p* < 0.05) for males and females, respectively (Figure 5C). In addition, the colorectal cancer ASRs for those countries in 2016 were calculated (Figure 5D). Of note, although Saudi Arabia had the lowest ASR, it presented the highest APC (for both genders) in contrast to the other countries in the comparison.

### 3.5. Distribution of Colorectal Cancer Stages

**Figure 6:**
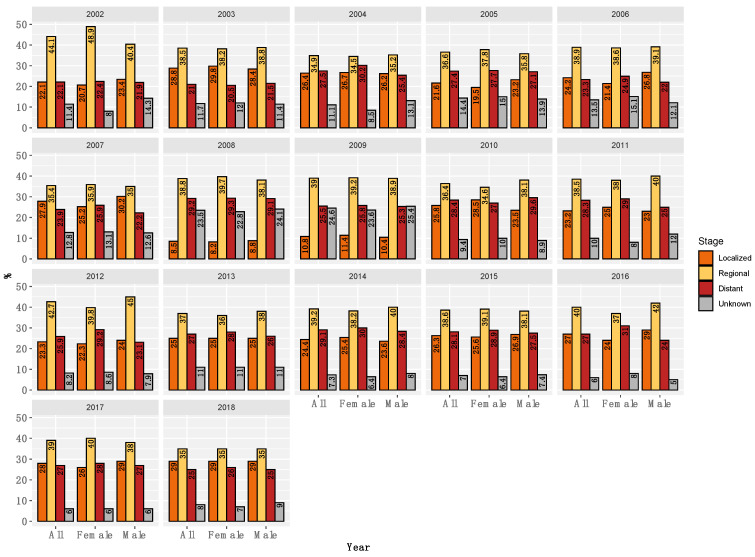
Distribution of CRC stages by year and gender. Percentages of each stage are displayed on the y-axis. CRC stages are color-coded (orange = localized, yellow = regional, dark red = distant, and grey = unknown). Each year in the period of 2001–2018 is shown at the top of each mini-plot. Genders (all, males, and females) are also denoted on the x-axis.

The colorectal cancer cases were subclassified by stage into localized, regional, distant, and unknown groups. For the period of 2002–2018, the regional stage remained constantly the dominant group regardless of gender (the mean percentages for all the years were 38.4%, 38.6%, and 38.3% for overall gender, males, and females, respectively. The distribution of all stages by gender is illustrated in Figure 6.

## 4. Discussion

The primary objective of this study was to elucidate the magnitude and direction of CRC incidence rate trends in the Saudi population by age, gender, and administrative region for the period of 2001–2018. The overall incidence of CRC is rising quickly, with the number of cases among Saudis having climbed by 335.6%. Throughout that period, CRC for both genders increased by 56.4% from 7.8% in 2001 to comprise 12.2% of all-cancers cases in 2018. This elevation was more noticeable in males compared to females (98% vs. 24.1%). Similarly, the CRC ASR had an overall increase of 152% for both genders, 178% for males and 126% for females, in that period. The AIR reached its highest point after the age of 70 years for both genders in most cases. Again, males had significantly higher AIR than females for the age groups above 65 years.

A thorough examination of the ASR revealed a few variations between geographical areas. Riyadh and the Eastern Region distinctly had the highest ASR among both genders, which peaked at 21.8 and 19.2 per 100 K population for males, and then, at 17.4 and 16.5 per 100 K population in the female cohort. This finding is probably not surprising given the fact that these two regions are the largest in the kingdom and home to more than 40% of the population. Additionally, the higher accessibility to advanced healthcare facilities in these regions, where a greater probability of detection of cases is anticipated, can partly account for this increase [16]. 

The CRC median age at diagnosis in our evaluation reached its peak at 60 years for males and 58 years in females. The increase in the median age at diagnosis was more pronounced in females (9.5%) than in males (5.3%), which seems to be consistent with Saudi Arabia’s present shift toward the acceptance of a more Westernized lifestyle, especially among women. In the United States (US), CRC has median age of diagnosis of 70 in males and 72 in females [17,18]. The younger median age at diagnosis among Saudis compared to other Western countries has also been noted in other types of cancer, such as breast cancer [19,20]. This is likely due to the fact that more than 60% of Saudi Arabia’s population is under 30 years old. Recently, the US Preventive Services Task Force (USPSTF) and American Cancer Society recommended that adults from the age of 45 years get screened for CRC, indicating the importance of targeting the younger generation [21]. In addition, men are diagnosed at a younger age when compared to women in the US. Interestingly, our analysis shows the opposite in Saudi Arabia. This could be explained by the notion that Saudi women have a lower tendency for seeking medical attention. 

Our prediction model to foresee the trends of colorectal cancer for the period of 2001–2018 revealed escalating trends with annual percentage changes (APCs) of 4.59% in males and 3.91% for females. Although Saudi Arabia’s ASR for CRC may appear low in comparison to various Western countries, our investigation of the APC of ASR in Saudi Arabia over a fifteen-year period (2002–2016) revealed an alarming rate for both genders (Saudi Arabia ranked first on the list with 4.1% and 3.4% for males and females, respectively). The significant increase in CRC incidence in Saudi Arabia is worth investigating.

The mean percentages of patients with regional (38.4%) and distant (26.2%) CRC in both genders are high in our analysis. In addition, the percentage of localized CRC in our analysis (23.7%) is less than that reported in the US (33.3%) [22]. This goes hand in hand with the previously reported reduced 5-year survival of 44.6% compared to 65.9% in the US [5]. Therefore, Saudi CRC patients are proportionally less diagnosed at an earlier stage. Instead, the cases are more diagnosed at later stages, and have poor survival. Diagnosis at a late stage is consistent with multiple reports and is likely due to the lack of an effective national CRC screening program [23,24,25]. 

An accelerated rise in the incidence of CRC is potentially a result of changes in multiple factors, such as obesity, physical activity, diet, smoking, socioeconomic status, longevity, screening measures, and treatment choices. Obesity, accompanied by physical inactivity, are among the most important behavioral factors contributing to the development of CRC [4]. According to a meta-analysis of 13 cohort studies, gaining 5 kg was linked to a 3% higher risk of CRC [26]. Additionally, abdominal fat and physical inactivity not only increase the risk of CRC but also reduce the likelihood of survival [27]. Furthermore, early-onset CRC risk has been associated with childhood obesity. [28]. Saudi Arabia has a high obesity rate (33%) with around one in every three Saudi men and two in every five Saudi women considered to be obese [25]. Unfortunately, as a consequence of growing urbanization, the prevalence of obesity is predicted to rise. In addition, reduced physical activity and sedentary lifestyles are frequent in Saudi Arabia and are largely likely attributable to cultural norms in some locations, as well as the excessively hot climate that lasts for several months during the year [29,30,31]. Effective efforts to reduce obesity and increase physical activity may have contributed to recent CRC prevention triumphs in developed nations such as Iceland, Japan, and the United States [32]. Exercise has been shown to lower precancerous lesions by roughly 15% and colon cancer risk by 20–25% [33,34]. 

It is known that the leading preventable cause of cancer mortality is smoking. According to a meta-analysis of 14 prospective cohort studies, compared to never smoking, smoking in the past and currently was linked to a worse prognosis for CRC. In addition, smoking termination was associated with enhanced overall and CRC-specific survival [35]. The estimated rate of smoking in Saudi Arabia climbed by 12.5% between 1990 and 2012 (the highest among the Gulf Cooperation Council nations), and it is anticipated to continue to rise [36]. 

Throughout the years, greater control of contagious and communicable diseases has led to a longer average life expectancy in Saudi Arabia. Between 1990 and 2014, there was a steady increase of 7.4% in the overall life expectancy [36]. It has been recognized that the incidence of most malignancies, including CRC, is thought to rise with advancing age and lifespan. This further suggests that an increase in the size of the older age groups in the Saudi population is likely associated with the rise in the number of cases documented.

By 2030, the numbers of new cases and annual fatalities from CRC are expected to rise by 60%, to about 2.2 million new cases and 1.1 million deaths. A key aspect of this increase is economic development and transitioning of the human development index (HDI). In countries going through significant developmental changes, incidence rates have a tendency to rise, along with rising HDI, indicating a strong correlation [1]. Between 1999 and 2018, Saudi Arabia’s HDI value increased by 22.8% [37]. The CRC incidence has been rising rapidly in nations going through a significant economic transformation and embracing a Western lifestyle [38].

It has been established that marital status is a significant predictor for cancer morbidity and mortality. Compared to married patients, single patients are more likely to experience delayed diagnosis, faster cancer growth, and slow recovery after surgery [39,40,41,42,43]. With the adoption of the “western” way of life, there is considerable evidence that the average age of first marriage has increased substantially [44]. According to one study in Saudi Arabia, patients who were not married had a 52% higher chance of developing CRC at an advanced stage and a 30% greater chance of dying from the disease compared to married patients [23].

This study, to our knowledge, is the longest analysis of CRC incidence trends in the Saudi population. However, information on mortality rates and survival, which are not presented here, is urgently needed. Considering that CRC-related deaths have fallen in multiple Western countries [45], it will be beneficial to investigate whether Saudi Arabia exhibits a similar behavioral pattern. Another limitation of this study is the possibility of bias due to the retrospective nature of data collection from multiple centers across multiple regions in the country. Also, molecular characteristics of tumors, which can provide deeper insight in our investigation, are lacking. Nonetheless, these caveats do not affect our findings, which provide comprehensive estimations of CRC trends in Saudi Arabia.

## 5. Conclusions

Saudi Arabia is experiencing a rise in the incidence of CRC, concomitant with changes in life expectancy, daily routines, and the adoption of Westernized lifestyle. Although the ASR for CRC may appear modest in Saudi Arabia compared to multiple Western nations, our investigation of the APC revealed a worrisome rate for both genders. Based on current data trends and lifestyle patterns, Saudi Arabia is anticipated to see an increase in CRC incidence (in both early at late onset) until it reaches a comparable ASR to that found in several Western countries. To reduce CRC incidence and death, long-term preventative actions and strategies should be used. The implementation of effective CRC screening before the age of 50 and for groups at risk is warranted. Personalized medicine should be given more attention, since predictive molecular diagnostics and targeted prevention can provide better treatment alternatives while also lowering costs. Furthermore, more efforts should be undertaken to combat tobacco use, as well as raise awareness of the importance of physical activity and a balanced diet.

## Figures and Tables

**Table 1:** Summary of colorectal cancer incidence parameters in Saudi Arabia.

Parameter	Sex	2001	2018	% Increase (2001–2018)
Total number of cases of all cancers among Saudis	Male	2875.0	6848.0	138.2
	Female	2741.0	8840.0	222.5
	Total	5616.0	15,688.0	179.3
Total number of colorectal cancer cases among Saudis	Male	222.0	1045.0	370.7
	Female	216.0	863.0	299.5
	Total	438.0	1908.0	335.6
% of colorectal cancer cases of all cancer cases among Saudis	Male	7.7	15.3	98.7
	Female	7.9	9.8	24.1
	Overall	7.8	12.2	56.4
Median age at colorectal cancer diagnosis	Male	57.0	60.0	5.3
	Female	53.0	58.0	9.4
Colorectal cancer ASR	Male	5.0	13.9	178.0
	Female	5.0	11.3	126.0
	Overall	5.0	12.6	152.0

Abbreviations: ASR = colorectal cancer overall age-standardized incidence rate.

**Table 2:** Trends of colorectal cancer incidence by age and sex (2001–2018).

Age Group	Sex Group	APC	95% CI
40–44	M	1.6	[0–3.3]
	F	1.9 *	[0.2–3.8]
45–49	M	3.2 *	[1.1–5.3]
	F	2.9 *	[0.3–5.7]
50–54	M	5.3 *	[3.4–7.1]
	F	5 *	[2.3–7.8]
55–59	M	4.9 *	[2.8–7.1]
	F	5 *	[2.7–7.4]
60–64	M	3.3 *	[1.5–5.1]
	F	4.8 *	[3.1–6.5]
65–69	M	4.2 *	[1.4–7.2]
	F	4.6 *	[2–7.1]
70–74	M	7.3 *	[5–9.7]
	F	5.4 *	[2.8–8]
75+	M	7.9 *	[5.3–10.7]
	F	3.7 *	[1.4–6.2]

Abbreviations: APC = annual percentage change; M = male; F = female; CI = confidence interval. * denotes *p* < 0.05.

**Table 3:** Trends of colorectal cancer incidence by administrative region and sex (2001–2018).

Region	Sex Group	APC	95% CI
Asir	M	6.8 *	[4.1–9.6]
	F	4 *	[0.3–8]
East	M	4.1 *	[2.7–5.5]
	F	4.3 *	[3.2–5.3]
Jazan	M	5.8 *	[2.4–9.4]
	F	4.6	[−3–13.1]
Madinah	M	3.5 *	[0.9–6.3]
	F	1.9	[−1.1–5.2]
Najran	M	3.3	[−2.2–9.1]
	F	0.7	[−6.3–8.2]
Qassim	M	6.6 *	[5–8.3]
	F	4.4 *	[0.8–8.2]
Tabuk	M	6 *	[1.3–11.1]
	F	2.8	[−1.2–7]
Baha	M	6.2 *	[0.3–12.6]
	F	4.8	[−0.4–10.5]
Hail	M	3.1	[−15.4–73]
	F	2.8	[−2.3–8.2]
Jouf	M	2.3	[–5–10]
	F	6.9 *	[0.1–14.2]
Makkah	M	2.5 *	[1–4]
	F	4.1 *	[2.1–6.1]
North	M	4.5	[−3.2–13.1]
	F	3.6	[−2.9–10.7]
Riyadh	M	4.8 *	[3.4–6.4]
	F	3.5 *	[2.6–4.3]

Abbreviations: APC = annual percentage change; M = male; F = female; CI = confidence interval. * denotes *p* < 0.05.

## Data Availability

Publicly available datasets were analyzed in this study. The data can be found here: https://nhic.gov.sa (accessed on 18 January 2023).

## Supplementary Materials

The following supporting information can be downloaded at: https://www.mdpi.com/article/10.3390/life13112198/s1, Table S1: Distribution of Saudi population, overall cancers, and CRC cases by gender and year (2001–2018).
